# Pancreatic β-Cell Death, Regeneration and Insulin Secretion: Roles of Poly(ADP-Ribose) Polymerase and Cyclic ADP-Ribose

**DOI:** 10.1080/15604280214485

**Published:** 2002

**Authors:** Shin Takasawa, Hiroshi Okamoto

**Affiliations:** 1 Department of Biochemistry Tohoku University Graduate School of Medicine Sendai 980-8575 Japan

## Abstract

In the early 1980s, we proposed a unifying model for β-cell damage (The OKAMOTO model), in which poly(ADP-ribose) synthetase/ polymerase (PARP) activation plays an essential role in the consumption of NAD^+^, which leads to energy depletion and necrotic cell death. In 1984, we demonstrated that the administration of PARP inhibitors to 90% depancreatized rats induces islet regeneration. From the regenerating islet-derived cDNA library we isolated *Reg* (Regenerating Gene) and demonstrated that Reg protein induces βcell replication via the Reg receptor and ameliorates experimental diabetes. More recently, we showed that the combined addition of IL-6 and dexamethasone induces the * Reg* gene expression in β-cells and that PARP inhibitors enhance the expression. In 1993, we found that cyclic ADP-ribose (cADPR), a product synthesized from NAD^+^, is a second messenger for intracellular Ca^+^ mobilization for insulin secretion by glucose, and proposed a novel mechanism of insulin secretion, the CD38-cADPR signal system.

Therefore, PARP inhibitors prevent β-cell necrosis, induce β-cell replication and maintain insulin secretion.

In this paper, we would like to present a perspective view based on our studies concerning cell death, cell regeneration, and cell function, especially on insulin-producing pancreatic βcells, in the processes of which poly(ADPribose) synthetase/polymerase (PARP) and cyclic ADP-ribose (cADPR) are functioning.


*Int. Jnl. Experimental Diab. Res.*, 3:79-96, 2002
© 2002 Taylor and Francis
1560-4284/02 $12.00 + .00

### Pancreatic ß-Cell Death, Regeneration and Insulin Secretion: Roles of Poly(ADP-Ribose) Polymerase and Cyclic ADP-Ribose

SHIN TAKASAWA AND HIROSHI OKAMOTO*

Department of Biochemistry, Tohoku University Graduate School of Medicine, Sendai 980-8575, Japan

*Received: xxx, 2001; In final form: xxx, 2001*

**In the early 1980s, we proposed a unifying model for ß-cell damage (The OKAMOTO model), in which poly(ADP-ribose) synthetase/polymerase (PARP) activation plays an essential role in the consumption of $NAD^+$, which leads to energy depletion and necrotic cell death. In 1984, we demonstrated that the administration of PARP inhibitors to 90% depancreatized rats induces islet regeneration. From the regenerating islet-derived cDNA library we isolated *Reg* (Regenerating Gene) and demonstrated that Reg protein induces ß-cell replication via the Reg receptor and ameliorates experimental diabetes. More recently, we showed that the combined addition of IL-6 and dexamethasone induces the *Reg* gene expression in ß-cells and that PARP inhibitors enhance the expression. In 1993, we found that cyclic ADP-ribose (cADPR), a product synthesized from $NAD^+$, is a second messenger for intracellular $Ca^{2+}$ mobilization for insulin secretion by glucose, and proposed a novel mechanism of insulin secretion, the CD38-cADPR signal system.**

**Therefore, PARP inhibitors prevent ß-cell necrosis, induce ß-cell replication and maintain insulin secretion.**

*Key Words:* Poly(ADP-ribose) synthetase/polymerase (PARP), Cyclic ADP-ribose, *Reg* gene, The OKAMOTO model, Necrosis

*Corresponding author: Department of Biochemistry, Tohoku University Graduate School of Medicine, Sendai 980-8575, Japan; e-mail: okamotoh@mail.cc.tohoku.ac.jp

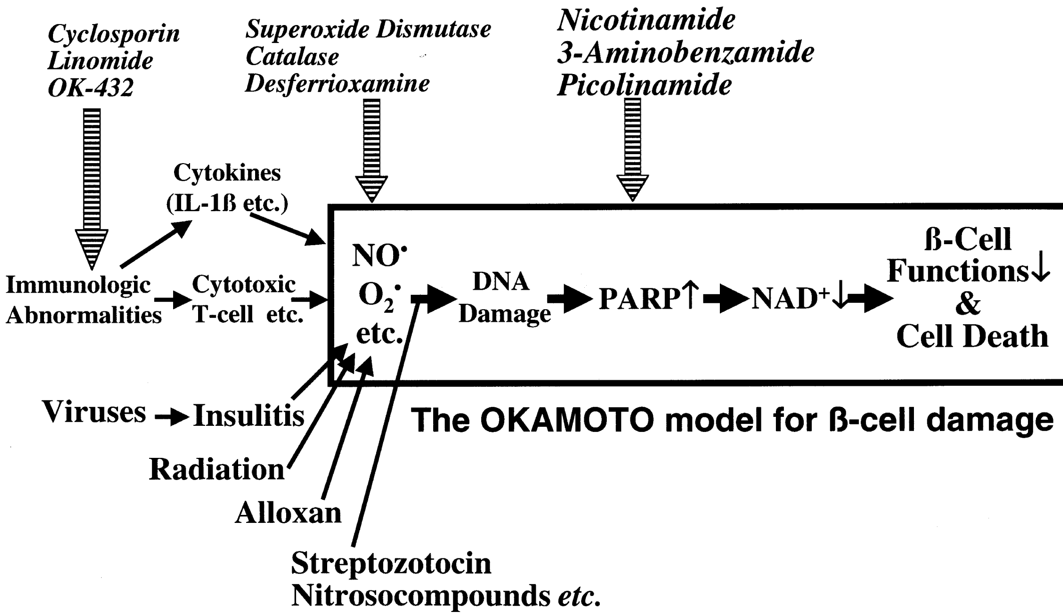

FIGURE 1
A unifying model for ß-cell damage and its prevention in toxin- or virus-induced and immune diabetes (The OKAMOTO model) (adopted from [8-10, 100]). The ß-cell damage is theoretically preventable through inhibition of the serial reactions, as indicated by shaded arrows. One method is by inhibiting abnormal immune reactions with immunomodulators such as cyclosporin, linomide and OK-432. Others include scavenging the radicals, which break DNA, by superoxide dismutase and other radical scavengers, and inhibiting the PARP by specific inhibitors such as nicotinamide, 3-aminobenzamide and picolinamide to prevent the decrease in the $NAD^+$ level. IL-1ß, interleukin-1ß. $NO^•$, nitric oxide.

In this paper, we would like to present a perspective view based on our studies concerning cell death, cell regeneration, and cell function, especially on insulin-producing pancreatic ß-cells, in the processes of which poly(ADP-ribose) synthetase/polymerase (PARP) and cyclic ADP-ribose (cADPR) are functioning.

### PANCREATIC ß-CELL DEATH BY PARP ACTIVATION

In 1981, we published two papers, one of which was concerned with *in vitro* experiments [1] and the other with *in vivo* experiments [2], describing that streptozotocin and alloxan induce DNA strand breaks and PARP in pancreatic islets, and proposed a unifying model for the action of diabetogenic agents, streptozotocin and alloxan, on pancreatic ß-cells [3]. Central to the model are breaks in the nuclear DNA of ß-cells, resulting from either an accumulation of free radicals or from alkylation of DNA. These breaks induce DNA repair involving the activation of PARP, which uses cellular $NAD^+$ as a substrate. As a result, the intracellular levels of $NAD^+$ fall dramatically. The fall in cellular $NAD^+$ inhibits cellular functions including insulin synthesis and secretion, and thus the ß-cell ultimately dies. Thus, this appears to be a suicide response for ß-cells to repair DNA. The $NAD^+$ depletion and the decrease in ß-cell functions, induced by alloxan and streptozotocin, were shown to be prevent-

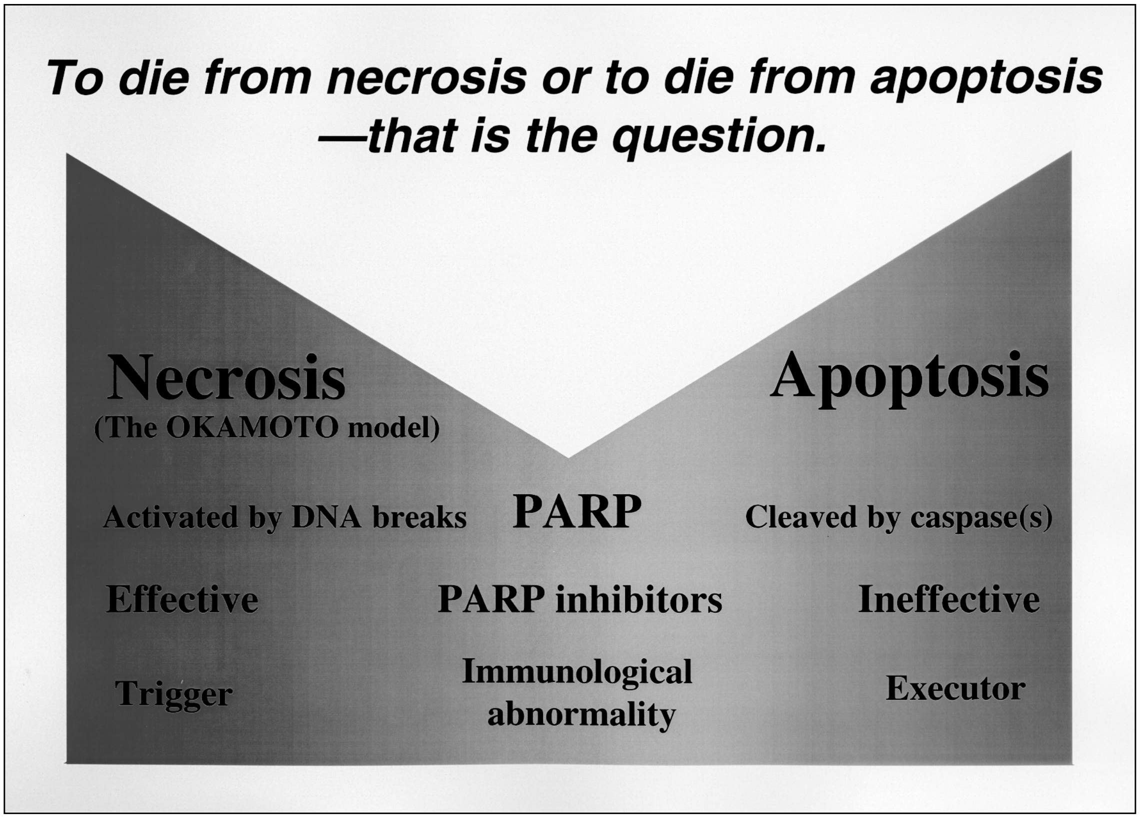

FIGURE 2
Comparison of two types of cell death, necrosis and apoptosis.

ed by radical scavengers such as superoxide dismutase and catalase and by PARP inhibitors [4, 5].

Interest in the model for the mechanism of action of alloxan and streptozotocin has been heightened by its possible extension to the effects of viruses and inflammation, especially immune-mediated events on ß-cells [3, 6-10]. Thus, since the early 1980s, we have thought that, although type 1 (insulin-dependent) diabetes can be caused by many different agents such as immunologic abnormalities, inflammatory tissue damage, and ß-cytotoxic chemical substances, the final pathway for the toxic agents is the same (Figure 1). This pathway involves DNA damage, PARP activation, and $NAD^+$ depletion. The fall in cellular $NAD^+$ inhibits cellular activities. Therefore, type 1 (insulin-dependent) diabetes is theoretically preventable by suppressing immune reactions, scavenging free radicals, and inhibiting PARP by nicotinamide and 3-aminobenzamide. Concerning nitric oxide (see Figure 1), we produced transgenic mice expressing nitric oxide synthase constitutively in pancreatic ß-cells and found that the ß-cell mass was markedly reduced and that the transgenic mice developed severe diabetes [11]. In 1999, using PARP deficient mice, three independent groups in Germany, Japan and U.S.A. provided irrefutable support for the model shown in Figure 1: PARP deficient mice were remarkably resistant to streptozotocin and did not show the ß-cell death [12-15]. More recently, many other tissues and cells have been reported to die by the same mechanism as in pancreatic ß-cell death [16-35].

The cell death caused by PARP activation described above is thought of in terms of necrosis [36]. In apoptotic cell death, PARP is cleaved by caspases and inactivated. Therefore, PARP inhibitors can be effective in preventing necrosis but ineffective in preventing apoptosis (Figure 2). "Whether to die from necrosis or to die from apoptosis" may depend on the severity and duration of the cell damage, differences in death signals, and the species of cells. A recent report from Bhardwaj's laboratory suggests that dendritic cells distinguish between two types of cell death, with necrosis providing a control that is critical for the initiation of immunity [37]. Therefore, immunological abnormalities, which are frequently observed in type 1 diabetes, may be triggered by the preceding necrotic cell death, and then cause apoptotic death of ß-cells.

### ß-CELL REGENERATION AND REG GENE

As described above, alloxan and streptozotocin diabetes can be prevented by PARP inhibitors. Concerning experimental diabetes, at the end of the 19th century von Mering and Minkowski in Strasbourg found that a dog became glycosuric and hyperglycemic by pancreatectomy. This observation stimulated many workers to try to isolate the active pancreatic principle as a possible treatment for diabetes. In 1984, we demonstrated that PARP inhibitors induce the regeneration of pancreatic ß-cells, thereby ameliorating surgical diabetes [38]. Male Wistar rats were 90% depancreatized, and nicotinamide or 3-aminobenzamide was injected intraperitoneally every day. The administration of PARP inhibitors ameliorated the surgical diabetes, and the islets in the remaining pancreases of rats that had received PARP inhibitors for 3 months were extremely large, and almost the entire areas of the enlarged islets were stained for insulin.

We isolated the regenerating islets and constructed a cDNA library. In screening the regenerating islet-derived cDNA library, we came across a novel gene expressed in regenerating islets. The cDNA had one large open reading frame which encoded a 165-amino acid protein. The deduced protein has a signal sequence. We propose to name the novel gene *Reg*, that is, **re**generating **g**ene, with the implication that the gene may be involved in islet regeneration [39]. We subsequently isolated human *REG* gene [39, 40]. Rat Reg protein increases [$^3$H]thymidine incorporation in rat islets, and mitosis was often observed [41]. We intraperitoneally injected rat Reg protein (1 mg/kg/day) to 90% depancreatized rats. On the 30th and 60th postoperative day, the fasting plasma glucose level of the rats receiving Reg protein was significantly lower than that of the 90% depancreatized control rats. After 2 months, almost all the islets of the 90% depancreatized control rats were destroyed. In contrast, the islets of the remaining pancreas in the Reg protein-treated rats were enlarged and the enlarged islets were densely and almost entirely stained for insulin [41]. These results indicate that Reg protein stimulates the regeneration and/or growth of pancreatic ß-cells, thereby ameliorating the surgical diabetes.

Recently, we isolated a Reg protein receptor cDNA from a λZAP II rat islet cDNA expression library [42]. The cDNA encoded a 919-amino acid protein, and the amino acid sequence suggested that the protein is a type II transmembrane protein with a long extracellular domain. We also isolated a human cDNA that shows over 97% amino acid identity to the rat homologue. The rat Reg receptor-expressing CHO cells bound rat Reg protein with high affinity ($Kd$ = 4.4 nM). The binding of $^{125}$I-labeled rat Reg protein was displaced by increasing the concentration of unlabeled rat Reg protein. Human REG protein, which shows 70% amino acid identity to rat Reg protein, also bound to the CHO cells (*Kd* = 14.0 nM), but higher concentrations of human REG protein were required for the displacement of the rat Reg protein. We established several cell lines of RINm5F cells overexpressing the Reg receptor. The cell lines showed significant increases in BrdU incorporation in the presence of 0.3-100 nM rat Reg protein. Moreover, the cell numbers were increased in response to Reg protein. The receptor mRNA was expressed in normal pancreatic islets, regenerating islets and a pancreatic ductal cell line, ARIP cells, that proliferate in a Reg protein-dependent manner.

The receptor mRNA expression was unchanged during islet regeneration [42]. This suggests that the regeneration and proliferation of pancreatic ß-cells are primarily regulated by the *Reg* gene expression. Accordingly, the transcriptional activation is of great importance in ß-cell regeneration. More recently, we found that *Reg* gene is activated by interleukin-6 (IL-6), dexamethasone, and PARP inhibitors [43]. The combined addition of IL-6 and dexamethasone increased the *Reg* mRNA level, and further addition of nicotinamide or 3-aminobenzamide increased the mRNA even more. Progressive deletion of the 5'-flanking region of rat *Reg* gene revealed that the region between nucleotides -81 and -70 is essential for the *Reg* gene promoter activity. The sequence is "TGCCCCTCCCAT". Similar GC box-like sequences were also observed in mouse and human *Reg* genes. The site-directed mutated luciferase construct "TGCCCCTAACAT" abolished the induction. The mutant ("TGCCCCGCCCAT"), which changed the sequence to a GC box, and the mutant ("TGCCCCACCCAT"), which changed the sequence of the rat *Reg* promoter to those of human *REG* genes, *REG Iα* [40] and *REG Iß* [44], showed the induction. In gel mobility shift assays (GMSA) with the GC box-like sequence, the intensity of the band, which was detected in the nuclear extracts of RINm5F cells treated with IL-6, dexamethasone and/or nicotinamide, was correlated with the luciferase activity [43]. The addition of $NAD^+$ to nuclear extracts attenuated the band, and nicotinamide and 3-aminobenzamide quenched the effect of $NAD^+$. These results suggest that PARP participates in the formation of the active transcriptional DNA/protein complex and that the formation of the active complex was inhibited by the poly(ADP-ribosyl)ation of nuclear proteins. The involvement of PARP in the active transcriptional complex was evidenced by the fact that the active transcriptional complex was stained by an anti-PARP antibody after GMSA analysis. The involvement of PARP in the active complex was further evidenced by the immunodepletion of PARP [43]. Southwestern experiments showed that a 113 kDa nuclear protein, the molecular weight of which corresponds to PARP, bound the GC box-like sequence. The band was recognized by the antibody to PARP. In fact, a purified recombinant PARP bound the *cis*-element. When nuclear extracts were incubated in the GMSA reaction in the presence of $[^{32}P]NAD^+$ and the reaction products were analyzed, only PARP was labeled. This suggests that PARP in the transcriptional complex auto-poly(ADP-ribosyl)ates itself.

Thus, as shown in Figure 3, inflammatory mediators, IL-6, and glucocorticoids induce the formation of an active transcriptional complex for *Reg* gene, in which PARP is involved, and the *Reg* gene transcription proceeds. On the other hand, during inflammation, superoxide ($O_2^{\bullet}$) and nitric oxide ($NO^{\bullet}$) are produced and cause DNA damage. In this case, PARP is activated by DNA nicks for the DNA repair. Then, PARP poly(ADP-ribosyl)ates PARP itself, the poly(ADP-ribose) chains on the PARP protein inhibit the formation of the active transcriptional complex, and the *Reg* gene transcription

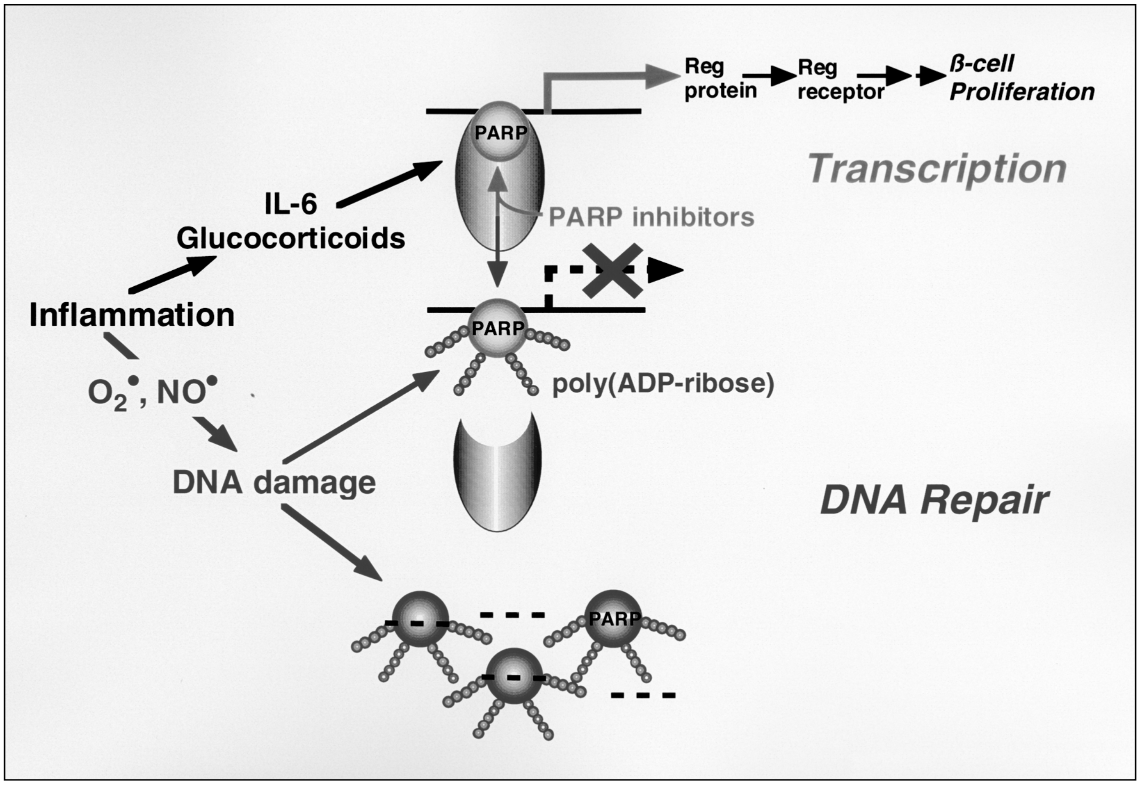

FIGURE 3
Representation of the unified role of PARP in the *Reg* gene transcription and DNA repair (adopted from [43]).

is stopped. In the presence of PARP inhibitors such as nicotinamide, the PARP is not poly(ADP-ribosyl)ated, the transcriptional complex is stabilized, and the *Reg* gene transcription proceeds. Therefore, PARP inhibitors keep PARP active as a transcription factor for ß-cell regeneration. This can account for the previous observation of islet regeneration in 90% depancreatized rats treated with PARP inhibitors [38] and also supports our previous proposition that the restriction of ß-cell replication is relieved by PARP inhibitors [6]. When DNA is massively damaged, PARP is rapidly activated to repair the DNA, as mentioned in the first part of this paper, and the complex for *Reg* gene transcription is not formed at all.

Recently, *Reg* and *Reg*-related genes have been isolated and revealed to constitute a multigene family, the *Reg* gene family [44-63]. Based on the primary structures of the Reg proteins, the members of the family are grouped into three subclasses, type I, II, III [45, 52]. Type I (and Type II) Reg proteins, about which we have discussed above, are expressed in regenerating islets [45]. Type III Reg proteins have also been suggested to be involved in cellular proliferation in intestinal cells, hepatic cells, and neuronal cells. In fact, a Cambridge group reported that mouse Reg III is a Schwann cell mitogen accompanying the regeneration of motor neurons [64], and a French group recently reported that Reg protein functions as a neurotrophic factor for motor neurons [65]. A Kyoto group reported that regenerating gene

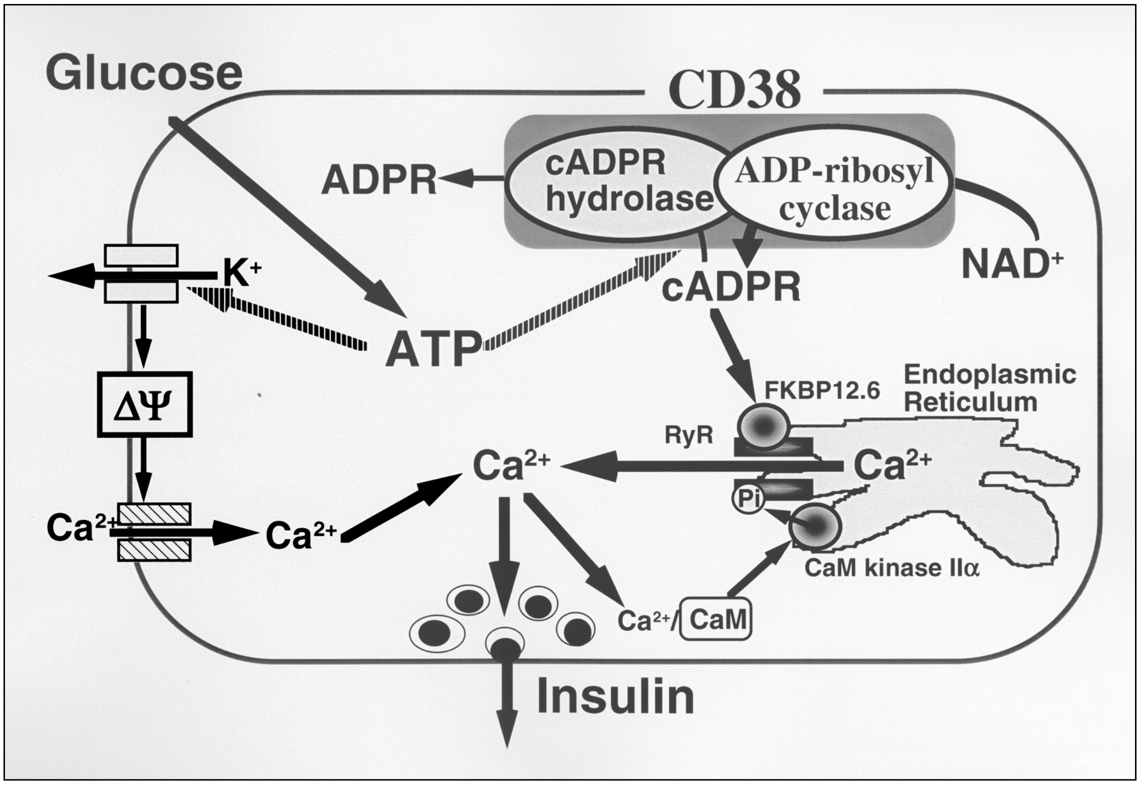

FIGURE 4
Insulin secretion by glucose stimulation in ß-cells (adopted from [100]). The insulin secretion via the CD38—cADPR signal system is shown in red. cADPR binds to FKBP12.6 to release $Ca^{2+}$, dissociating FKBP12.6 from RyR [81]. CaM kinase II phosphorylates RyR to sensitize and activate the $Ca^{2+}$ channel (*Pi*, phosphorylation of RyR by CaM kinase II) [84]. $Ca^{2+}$, released from intracellular stores and/or supplied from extracellular sources, further activates CaM kinase II and amplifies the process. In this way, $Ca^{2+}$-induced $Ca^{2+}$ release (CICR) can be explained. The conventional insulin secretion mechanism by $Ca^{2+}$ influx from extracellular sources [69] is shown on in black. ADPR, ADP-ribose.

protein may mediate the gastric mucosal proliferation induced by hypergastrinemia in rats [66-68]. The expression of Reg protein receptor mRNA was also detected in liver, kidney, stomach, small intestine, colon, adrenal gland, pituitary gland, and brain [42], suggesting the possible involvement of the Reg protein-Reg receptor signal system in a variety of cell types other than pancreatic ß-cells.

### THE CD38-cADPR SIGNAL SYSTEM FOR INSULIN SECRETION IN ß-CELLS

cADPR is synthesized from $NAD^+$, and our results have shown that cADPR is a second messenger for intracellular $Ca^{2+}$ mobilization for insulin secretion in pancreatic ß-cells. Therefore, decreases in the $NAD^+$ level (see Figure 1) can cause decreases in cADPR and then in insulin secretion.

Glucose induces an increase in the intracellular $Ca^{2+}$ concentration in pancreatic ß-cells of the islets of *Langerhans* to cause the secretion of insulin. This increase in the $Ca^{2+}$ concentration was first explained in 1984 by the hypothesis of Ashcroft et al. of Oxford University [69], in which $Ca^{2+}$ is provided extracellularly. That is, millimolar concentrations of ATP, produced in the process of glucose metabolism,

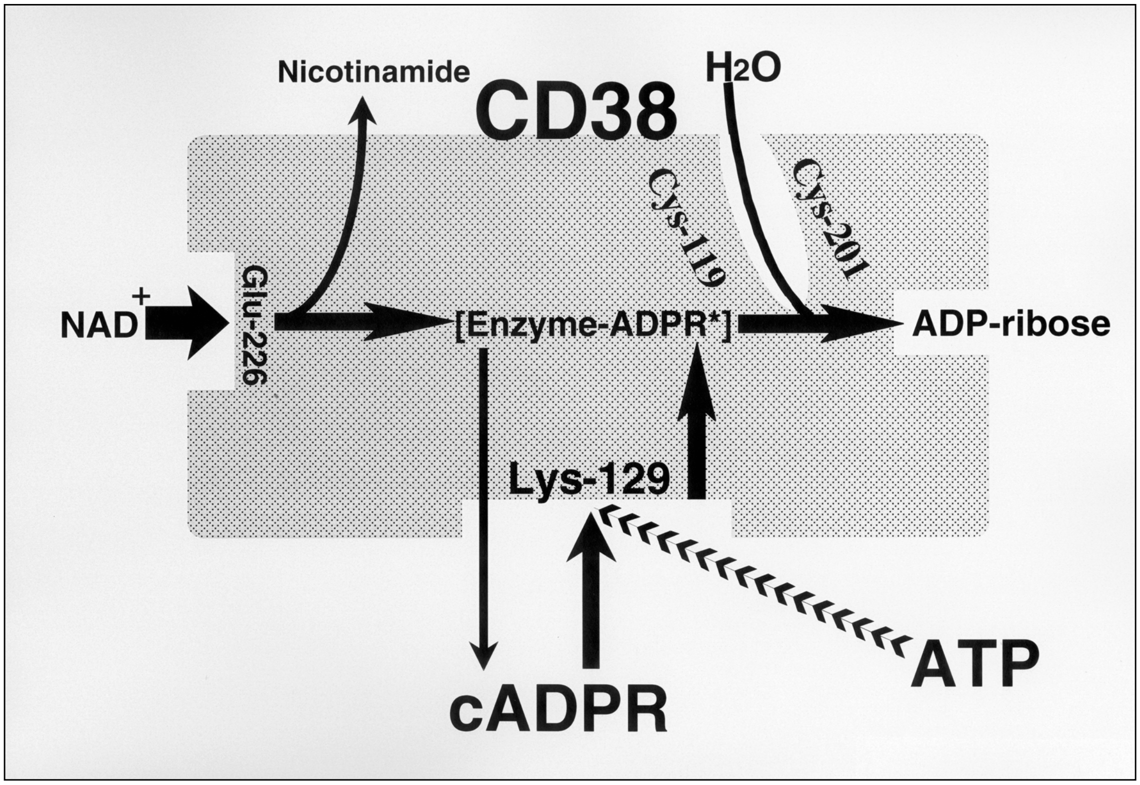

FIGURE 5
Roles of amino acid residues of CD38 in enzymic activities (adapted from [76]). Glu-226 is essential for ADP-ribosyl cyclase. Cys-119 and Cys-201 are essential for cADPR hydrolase [77]. Lys-129 is cADPR binding site and essential for cADPR hydrolase [78]. ATP competes with cADPR for the binding site (Lys-129), inhibiting the cADPR hydrolysis to accumulate cADPR. [Enzyme-ADPR*] is proposed as an enzyme-stabilized ADP-ribosyl oxocarbonium ion intermediate [76].

inhibit the potassium channel, inducing membrane depolarization and the opening of the voltage-dependent $Ca^{2+}$ channels. In 1993, we proposed another model of insulin secretion by glucose via cADPR-mediated $Ca^{2+}$ mobilization from an intracellular $Ca^{2+}$ pool, the endoplasmic reticulum [70], as shown in Figure 4. That is, ATP inhibits the cADPR hydrolase of CD38, causing the accumulation of cADPR, which acts as a second messenger for $Ca^{2+}$ mobilization from the endoplasmic reticulum for insulin secretion. The first important issue is whether the accumulation of cADPR is actually caused by glucose stimulation in pancreatic islets. We incubated normal rat (Wistar) and mouse (C57BL/6J) islets with low (2.8 mM) glucose and high (20 mM) glucose, and assayed the cADPR content in the islets by radioimmunoassay using an anti-cADPR antibody. The cADPR content of islets incubated with high glucose was increased within 5 min, whereas the cADPR content of islets incubated with low glucose was not [71]. Next, we used rat pancreatic islet microsomes as a cell-free system to study $Ca^{2+}$ release and found that cADPR released $Ca^{2+}$ from islet microsomes, as indicated by the observed prompt increase in fluo 3 fluorescence [70, 71]. Inositol 1,4,5-trisphosphate ($IP_3$) did not cause the release of $Ca^{2+}$, and at this point, the islet microsomes were still responsive to cADPR. We then used rat cerebellum microsomes. $IP_3$ caused a release of $Ca^{2+}$

from cerebellum microsomes. cADPR also caused a release of $Ca^{2+}$. Heparin, an inhibitor of $IP_3$ binding to its receptor, blocked the $IP_3$-induced $Ca^{2+}$ release from cerebellum microsomes, but did not block the cADPR-induced $Ca^{2+}$ release. These results indicate that islet microsomes respond to cADPR but not to $IP_3$. In contrast, cerebellum microsomes respond to both cADPR and $IP_3$, but cADPR induces the $Ca^{2+}$ release via a different mechanism than that utilized by $IP_3$. We then examined the effect of cADPR on insulin secretion using digitonin-permeabilized pancreatic islets. cADPR as well as $Ca^{2+}$ induced insulin secretion, but $IP_3$ did not. The combined addition of cADPR and $Ca^{2+}$ did not induce significantly more insulin secretion than the addition of cADPR or $Ca^{2+}$ alone. The cADPR-induced insulin secretion was inhibited by the addition of EGTA. These results suggested that the cADPR-induced insulin secretion was mediated by $Ca^{2+}$ mobilization from islet microsomes [70]. Thus, we proposed that glucose stimuli induce cADPR formation from $NAD^+$. cADPR then mobilizes $Ca^{2+}$ from the endoplasmic reticulum, serving as a second messenger for insulin secretion.

The next question is why the glucose stimulus induces the formation of cADPR. CD38 is a 300-amino acid protein and was first recognized as a leukocyte antigen. We found that CD38 is expressed in a variety of tissues including pancreatic ß-cells [72, 73]. We and others have found that CD38 has both ADP-ribosyl cyclase, synthesizing cADPR from $NAD^+$, and cADPR hydrolase to produce ADP-ribose [72, 74, 75]. We purified human CD38 protein and found that millimolar concentrations of ATP inhibit the cADPR hydrolase activity of CD38, competing with the substrate, cADPR [76]. The competitive inhibition of the cADPR hydrolysis by ATP suggests that ATP and cADPR bind to the same site of CD38. We then labeled the purified CD38 with an ATP analogue, 5'-*p*-fluorosulfonylbenzoyladenosine, and identified the binding site for ATP and/or cADPR as the lysine-129 of CD38 [76]. From these results and other available evidence, we proposed that CD38 catalyzes the formation of cADPR from $NAD^+$ and also the hydrolysis of cADPR to ADP-ribose. As shown in Figure 5, lysine-129 of CD38 is the cADPR binding site, and ATP competes with cADPR for the binding site, resulting in the inhibition of the hydrolysis of cADPR and then, in the accumulation of cADPR [76]. Cysteine-119 and Cysteine-201 are essential for the hydrolase reaction [77], and glutamic acid-226 for the $NAD^+$ binding [78].

cADPR has been thought to activate the ryanodine receptor to release $Ca^{2+}$ from the intracellular stores, the endoplasmic reticulum [70, 79, 80]. We have shown that the type 2 ryanodine receptor is expressed in rat pancreatic islets [71]. Our experiments indicated that cADPR did not bind directly to the ryanodine receptor but may act on the receptor through a mediator such as FK506-binding protein 12.6, FKBP12.6, to release $Ca^{2+}$. FK506 is one of the most widely used immunosuppressive agents. The cellular target for FK506 is thought to be FKBP12 and FKBP12.6. Rat FKBP12 is composed of 108 amino acids and is highly conserved among human, mouse, bovine, and rabbit FKBP12. Rat FKBP12.6 is also a 108-amino acid protein as are human and bovine FKBP12.6. Rat islet microsomes contained FKBP12.6, but did not contain FKBP12. It is of great interest that cADPR was found to bind to FKBP12.6 at a *Kd* value of 35 nM. The binding of radiolabeled cADPR was inhibited by cold FK506 as well as cADPR and neither structurally nor functionally related analogues of cADPR inhibited the cADPR binding to FKBP12.6 [81]. These results indicate that FKBP12.6 acts as a cADPR-binding protein and strongly suggest that cADPR is the actual ligand for FKBP12.6 since FK506 does not normally exist in mammalian cells. FKBP12.6 occurs in rat islet microsomes. However, when rat islet microsomes were treated with cADPR, FKBP12.6 dissociated from the microsomes and moved to the supernatant, releasing $Ca^{2+}$ from the intracellular stores [81]. From these results together with other experiments, it is strongly suggested that, when cADPR binds to FKBP12.6 in the ryanodine receptor and causes the dissociation of FKBP12.6 from the ryanodine receptor to form the FKBP12.6-cADPR complex, the channel activity of the ryanodine receptor is thereby increased to release $Ca^{2+}$ from the endoplasmic reticulum. As you can also see in Figure 4, when FK506 is present, cADPR cannot act on the ryanodine receptor to release $Ca^{2+}$ and the glucose-induced insulin secreting machinery ceases to function. In fact, when FK506 was used as an immuno-suppressant in kidney transplantation, hyperglycemia was observed in 20-35 per cent of the recipients [82, 83]. The diabetogenic side effect of FK506 may be explained by the mechanism shown in Figure 4. Furthermore, in the presence of calmodulin, islet microsomes were sensitized to cADPR at much lower concentrations for $Ca^{2+}$ release, and the $Ca^{2+}$ release was greatly increased [84, 85]. These results and other available evidence suggest that the cADPR-mediated $Ca^{2+}$ mobilization for insulin secretion is achieved by the calmodulin-activated CaM kinase II. Possibly, the activated kinase phosphorylates the ryanodine receptor to sensitize the $Ca^{2+}$ channel for the cADPR signal.

To verify a novel mechanism of insulin secretion, the CD38—cADPR signal system, we created CD38 knockout mice [86]. The pancreatic islets of CD38 knockout mice showed almost no ADP-ribosyl cyclase activity. The glucose-induced increase in the intracellular $Ca^{2+}$ concentration was severely impaired in the knockout mouse islets, and the glucose-induced insulin secretion was severely decreased. The knockout islets, however, responded normally to the extracellular $Ca^{2+}$ influx stimulants tolbutamide and KCl to secrete insulin [86, 87]. This suggests that the CD38—cADPR signal system functions in the $Ca^{2+}$ mobilization from intracellular $Ca^{2+}$ stores. The paradigm of insulin secretion based on the CD38—cADPR signal system, so far described, relies on a wide body of evidence obtained in rat and mouse. Our recent results indicate that the CD38—cADPR signal system functions in insulin secretion in man. We identified a missense mutation in the CD38 gene [88, 89] in Japanese diabetic patients [90]. The resulting CD38 protein showed altered catalytic activities, with a decreased production of cADPR. Furthermore, circulating anti-CD38 autoantibodies have been detected in 10-14% of Japanese [91] as well as Caucasian diabetic patients [92-94]. The autoantibody altered the enzymic activity of islet CD38 and insulin secretion *in vitro*. These findings strongly suggest that the CD38—cADPR signal system functions in insulin secretion by glucose in man.

The CD38—cADPR signal system for insulin secretion is different from the conventional hypothesis [69] in which $Ca^{2+}$ influx from extracellular sources plays a role in insulin secretion by glucose. Furthermore, the CD38—cADPR signal system is also different from the hypothesis proposed by Berridge and Irvine of Cambridge University [95], in which $IP_3$ induces $Ca^{2+}$ release from the intracellular pool, the endoplasmic reticulum. In this context, the CD38—cADPR signal system was the focus of intense debate [96-100]. Controversial results were reported using diabetic ß-cells such as *ob/ob* mouse islets and RINm5F cells, which have been traditionally used for studying insulin secretion in Europe and U.S.A. We revealed that the $Ca^{2+}$ release responses of these diabetic ß-cell microsomes were quite different from those of normal islet microsomes [71]. Microsomes from normal C57BL mouse islets released $Ca^{2+}$ in response to cADPR but scarce-

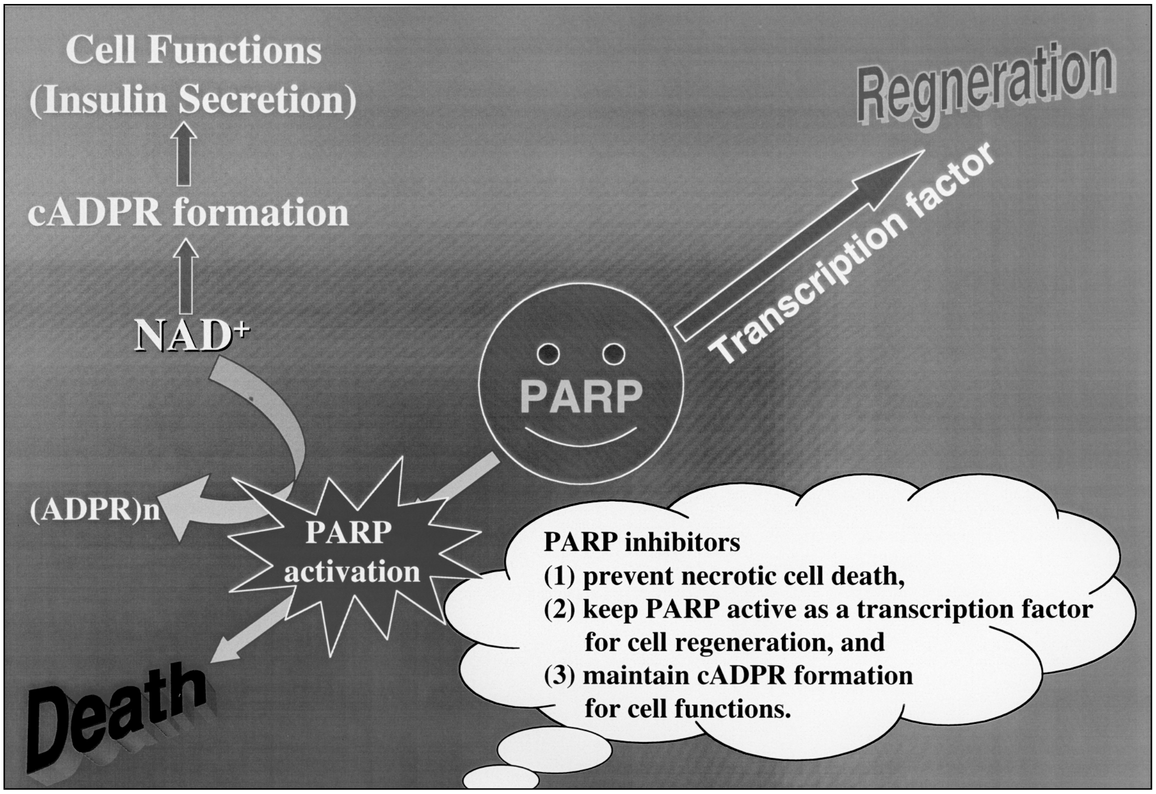

FIGURE 6
Roles of PARP inhibitors for cell death, regeneration, and functioning.

ly in response to $IP_3$. This response to cADPR was completely attenuated by the prior addition of 8-amino-cADPR, an antagonist of cADPR [101]. In contrast to normal islet microsomes, *ob*/*ob* mouse islet microsomes released only a small amount of $Ca^{2+}$ by cADPR but released much $Ca^{2+}$ by $IP_3$. RINm5F cell microsomes responded well to $IP_3$ to release $Ca^{2+}$ but did not respond to cADPR. RINm5F cells are rat insulinoma-derived immortal cells and show almost no glucose-induced insulin secreting ability. Furthermore, the CD38 mRNA level was significantly decreased in *ob*/*ob* islets [71], and in RINm5F cells, CD38 mRNA was not expressed [73]. These results indicate that the CD38-cADPR signal system for insulin secretion is used under normal physiological conditions, and is replaced by the $IP_3$ system in diabetic ß-cells such as *ob*/*ob* mouse islets and RINm5F cells. In fact, Balb/c mouse islets showed distinct increases in intracellular cADPR, $Ca^{2+}$, and insulin secretion by glucose [102], and MIN6 cells showed a dramatic $Ca^{2+}$ mobilization in response to cADPR via the ryanodine receptor despite the fact that no response to $IP_3$ was observed [80].

Although $IP_3$ has been thought to be a second messenger for $Ca^{2+}$ mobilization from intracellular stores, as described above, cADPR induces $Ca^{2+}$ release from pancreatic islet microsomes but $IP_3$ does not. In cerebellum microsomes, both cADPR and $IP_3$ induced $Ca^{2+}$ release. Therefore, cells can utilize two second messengers, $IP_3$ and cADPR, for $Ca^{2+}$ mobilization, depending on the species of cells as well as differences in cellular conditions, physiological or pathological, performing a variety of cellular functions. Recently, various physiological phenomena from animal to plant cells become understandable in terms of this novel signal system [103-127]. In pancreatic acinar cells of CD38-knockout mice, the acetylcholine-induced $Ca^{2+}$ oscillation was greatly reduced or completely disappeared under a physiological concentration of acetylcholine [126]. Furthermore, acetylcholine induced the cADPR formation in normal acinar cells, but not in CD38 knockout acinar cells. The $IP_3$ formation was very small in the presence of a physiological concentration of acetylcholine and there was no difference between normal and CD38 knockout cells. Probably, acetylcholine induces the cADPR formation via the G-protein coupled CD38 system [128]. In pancreatic ß-cells, glucose is metabolized and induces the CD38-cADPR system to secrete insulin. In many other cells, hormones and neurotransmitters may regulate the CD38-cADPR signal system in a receptor-coupled manner, such as in a G-protein-coupled manner, to express various physiological responses.

### CONCLUSION AND FUTURE PROSPECTS

In the first part of this paper, we described that PARP activation causes NAD+ depletion to form poly(ADP-ribose), resulting in necrotic ß-cell death. More recently, accumulating evidence has confirmed that the mechanism proposed for ß-cell death is involved in the process of the cell death of many tissues and cells. In the second part, we described ß-cell regeneration and *Reg* gene, showing that PARP acts as a transcription factor for *Reg* gene, and that the active transcriptional complex for *Reg* gene is not formed when PARP is activated and auto-poly(ADP-ribosyl)ated. Recently, Reg proteins have been shown to be a growth factor for Schwann cells, neuronal cells, and gastrointestinal cells. In the last part, we described that the cADPR formation from $NAD^+$ is essential for insulin secretion by glucose in ß-cells. Recently, various physiological phenomena from animal to plant cells become understandable in terms of a novel signal system, the CD38-cADPR signal system. Therefore, the inhibition of the PARP activity by PARP inhibitors results in at least three important events in the cell: PARP inhibitors prevent the necrotic cell death, keep PARP active as a transcription factor for cell regeneration, and maintain the formation of a second messenger, cADPR, to achieve the cell function (Figure 6).

***Acknowledgements***
The authors thank Brent Bell for valuable assistance in preparing the manuscript for publication. This work was supported in part by grants-in-aid from the Ministry of Education, Culture, Sports, Science, Sports and Technology, Japan.

### REFERENCES

1. Yamamoto, H., Uchigata, Y. and Okamoto, H. (1981) Streptozotocin and alloxan induce DNA strand breaks and poly(ADP-ribose) synthetase in pancreatic islets, *Nature*, **294**, 284-286.
2. Yamamoto, H., Uchigata, Y. and Okamoto, H. (1981) DNA strand breaks in pancreatic islets by *in vivo* administration of alloxan or streptozotocin, *Biochem. Biophys. Res. Commun.*, **103**, 1014-1020.
3. Okamoto, H. (1981) Regulation of proinsulin synthesis in pancreatic islets and a new aspect to insulin-dependent diabetes, *Mol. Cell. Biochem.*, **37**, 43-61.
4. Uchigata, Y., Yamamoto, H., Kawamura, A. and Okamoto, H. (1982) Protection by superoxide dismutase, catalase, and poly(ADP-ribose) synthetase inhibitors against alloxan- and streptozotocin-induced islet DNA strand breaks and against the inhibition of proinsulin synthesis, *J. Biol. Chem.*, **257**, 6084-6088.
5. Uchigata, Y., Yamamoto, H., Nagai, H. and Okamoto, H. (1983) Effect of poly(ADP-ribose) synthetase inhibitor administration to rats before and after injection of alloxan and streptozotocin on islet proinsulin synthesis, *Diabetes*, **32**, 316-318.

6. Okamoto, H. (1985) Molecular basis of experimental diabetes: Degeneration, oncogenesis and regeneration of pancreatic B-cells of islets of Langerhans, *BioEssays,* **2**, 15-21.

7. Okamoto, H., Yamamoto, H., Takasawa, S., Inoue, C., Terazono, K., Shiga, K. and Kitagawa, M. (1988) Molecular mechanism of degeneration, oncogenesis and regeneration of pancreatic B-cells of islets of Langerhans. In: *Lessons from Animal Diabetes II*, Edited by Shafrir, E. and Renold, A. E., pp. 149-157. London, John Libbey & Company Ltd.

8. Okamoto, H. (1990) The molecular basis of experimental diabetes. In: *Molecular Biology of the Islets of Langerhans*, Edited by Okamoto, H., pp. 209-231. Cambridge, Cambridge University Press.

9. Okamoto, H., Takasawa, S. and Tohgo, A. (1995) New aspects of the physiological significance of NAD, poly ADP-ribose and cyclic ADP-ribose, *Biochimie,* **77**, 356-363.

10. Okamoto, H. (1996) The OKAMOTO model for B-cell damage: recent advances. In: *Lessons from Animal Diabetes VI*, Edited by Shafrir, E., pp. 97-111. Boston, Birkhäuser.

11. Takamura, T., Kato, I., Kimura, N., Nakazawa, T., Yonekura, H., Takasawa, S. and Okamoto, H. (1998) Transgenic mice overexpressing type 2 nitric oxide synthase in pancreatic ß cells develop insulin-dependent diabetes without insulitis, *J. Biol. Chem.,* **273**, 2493-2496.

12. Charron, M. J. and Bonner-Weir, S. (1999) Implicating PARP and NAD+ depletion in type I diabetes, *Nature Med.,* **5**, 269-270.

13. Burkart, V., Wang, Z. Q., Radons, J., Heller, B., Herceg, Z., Stingl, L., Wagner, E. F. and Kolb, H. (1999) Mice lacking the poly(ADP-ribose) polymerase gene are resistant to pancreatic beta-cell destruction and diabetes development induced by streptozocin, *Nature Med.*, **5**, 314-319.

14. Masutani, M., Suzuki, H., Kamada, N., Watanabe, M., Ueda, O., Nozaki, T., Jishage, K., Watanabe, T., Sugimoto, T., Nakagama, H., Ochiya, T. and Sugimura, T. (1999) Poly(ADP-ribose) polymerase gene disruption conferred mice resistant to streptozotocin-induced diabetes, *Proc. Natl. Acad. Sci. USA,* **96**, 2301-2304.

15. Pieper, A. A., Brat, D. J., Krug, D. K., Watkins, C. C., Gupta, A., Blackshaw, S., Verma, A., Wang, Z. Q. & Snyder, S. H. (1999) Poly(ADP-ribose) polymerase-deficient mice are protected from streptozotocin-induced diabetes, *Proc. Natl. Acad. Sci. USA,* **96**, 3059-3064.

16. Eliasson, M. J., Sampei, K., Mandir, A. S., Hurn, P. D., Traystman, R. J., Bao, J., Pieper, A., Wang, Z. Q., Dawson, T. M., Snyder, S. H. and Dawson, V. L. (1997) Poly(ADP-ribose) polymerase gene disruption renders mice resistant to cerebral ischemia, *Nature Med.*, **3**, 1089-1095.

17. Szabó, C., Cuzzocrea, S., Zingarelli, B., O'Connor, M. and Salzman, A. L. (1997) Endothelial dysfunction in a rat model of endotoxic shock: Importance of the activation of poly(ADP-ribose) synthetase by peroxynitrite, *J. Clin. Invest.*, **100**, 723-735.

18. Zingarelli, B., Salzman, A. L. and Szabo, C. (1998) Genetic disruption of poly (ADP-ribose) synthetase inhibits the expression of P-selectin and intercellular adhesion molecule-1 in myocardial ischemia/reperfusion injury, *Circ. Res.*, **83**, 85-94.

19. Szabó, C., Virág, L., Cuzzocrea, S., Scott, G. S., Hake, P., O'Connor, M. P., Zingarelli, B., Salzman, A. and Kun, E. (1998) Protection against peroxynitrite-induced fibroblast injury and arthritis development by inhibition of poly(ADP-ribose) synthetase, *Proc. Natl. Acad. Sci. USA*, **95**, 3867-3872.

20. Mandir, A. S., Przedborski, S., Jackson-Lewis, V., Wang, Z. Q., Simbulan-Rosenthal, C. M., Smulson, M. E., Hoffman, B. E., Guastella, D. B., Dawson, V. L. and Dawson, T. M. (1999) Poly(ADP-ribose) polymerase activation mediates 1-methyl-4-phenyl-1,2,3,6-tetrahydropyridine (MPTP)-induced parkinsonism, *Proc. Natl. Acad. Sci. USA*, **96**, 5774-5779.

21. Stern, Y., Salzman, A., Cotton, R. T. and Zingarelli, B. (1999) Protective effect of 3-aminobenzamide, an inhibitor of poly(ADP-ribose) synthetase, against laryngeal injury in rats, *Am. J. Resir. Crit. Care Med.*, **160**, 1743-1749.

22. Bowes, J., McDonald, M. C., Piper, J. and Thiemermann, C. (1999) Inhibitors of poly(ADP-ribose) synthetase protect rat cardiomyocytes against oxidant stress, *Cardiovasc. Res.*, **41**, 126-134.

23. Filipovic, D. M., Meng, X., and Reeves, W. B. (1999) Inhibition of PARP prevents oxidant-induced necrosis but not apoptosis in LLC-PK1 cells, *Am. J. Physiol.*, **277**, F428-F436.

24. Oliver, F. J., Ménissier-de Murcia, J., Nacci, C., Decker, P., Andriantsitohaina, R., Muller, S., de la Rubia, G., Stoclet, J. C. and de Murcia, G. (1999) Resistance to endotoxic shock as a consequence of defective NF-κB activation in poly (ADP-ribose) polymerase-1 deficient mice, *EMBO J.*, **18**, 4446-4454.

25. Zingarelli, B., Szabó, C. and Salzman, A.L. (1999) Blockade of poly(ADP-ribose) synthetase inhibits neutrophil recruitment, oxidant generation, and mucosal injury in murine colitis, *Gastroenterology*, **116**, 335-345.

26. Tsao, B. P., Cantor, R. M., Grossman, J. M., Shen, N., Teophilov, N. T., Wallace, D. J., Arnett, F. C., Hartung, K., Goldstein, R., Kalunian, K. C., Hahn, B. H. and Rotter, J. I. (1999) PARP alleles within the linked chromosomal region are associated with systemic lupus erythematosus, *J. Clin. Invest.*, **103**, 1135-1140.

27. Plaschke, K., Kopitz, J., Weigand, M. A., Martin, E. and Bardenheuer, H. J. (2000) The neuroprotective effect of cerebral poly(ADP-ribose)polymerase inhibition in a rat model of global ischemia, *Neurosi. Lett.*, **284**, 109-112.

28. Ducrocq, S., Benjelloun, N., Plotkine, M., Ben-Ari, Y. and Charriaut-Marlangue, C. (2000) Poly(ADP-ribose) synthase inhibition reduces ischemic injury and inflammation in neonatal rat brain, *J. Neurochem.*, **74**, 2504-2511.

29. Mandir, A. S., Poitras, M. F., Berliner, A. R., Herring, W. J., Guastella, D. B., Feldman, A., Poirier, G. G., Wang, Z. Q., Dawson, T. M. and Dawson, V. L. (2000) NMDA but not non-NMDA excitotoxicity is mediated by Poly(ADP-ribose) polymerase, *J. Neurosci.*, **20**, 8005-8011.

30. Pieper, A. A., Walles, T., Wei, G., Clements, E. E., Verma,

A., Snyder, S. H. and Zweier, J. L. (2000) Myocardial postischemic injury is reduced by polyADPribose polymerase-1 gene disruption, *Mol. Med.*, **6,** 271-282.

31. Liaudet, L., Soriano, F. G., Szabó, E., Virag, L., Mabley, J. G., Salzman, A. L. and Szabó, C. (2000) Protection against hemorrhagic shock in mice genetically deficient in poly(ADP-ribose)polymerase, *Proc. Natl. Acad. Sci. USA*, **97,** 10203-10208.
32. McDonald, M. C., Mota-Filipe, H., Wright, J. A., Abdelrahman, M., Threadgill, M. D., Thompson, A. S. and Thiemermann, C. (2000) Effects of 5-aminoisoquinolinone, a water-soluble, potent inhibitor of the activity of poly (ADP-ribose) polymerase on the organ injury and dysfunction caused by haemorrhagic shock, *Br. J. Pharmacol.*, **130,** 843-850.
33. Jijon, H. B., Churchill, T., Malfair, D., Wessler, A., Jewell, L. D., Parsons, H. G. and Madsen, K. L. (2000) Inhibition of poly(ADP-ribose) polymerase attenuates inflammation in a model of chronic colitis, *Am. J. Physiol.*, **279,** G641-G651.
34. Martin, D. R., Lewington, A. J., Hammerman, M. R. and Padanilam, B. J. (2000) Inhibition of poly(ADP-ribose) polymerase attenuates ischemic renal injury in rats, *Am. J. Physiol.*, **279,** R1834-R1840.
35. Soriano, F. G., Virág, L., Jagtap, P., Szabó, E., Mabley, J. G., Liaudet, L., Marton, A., Hoyt, D. G., Murthy, K. G. K., Salzman, A. L., Southan, G. J. and Szabó, C. (2001) Diabetic endothelial dysfunction: the role of poly(ADP-ribose) polymerase activation, *Nature Med.*, **7,** 108-113.
36. Germain, M., Scovassi, I. and Poirier, G. G. (2000) Role of poly(ADP-ribose) polymerase in apoptosis. In: *Cell Death—The Role of Poly(ADP-ribose) polymerase—*, Edited by Szabó, C., pp. 209-225. Boca Raton, CRC Press.
37. Sauter, B., Albert, M. L., Francisco, L., Larsson, M., Somersan, S. and Bhardwaj, N. (2000) Consequences of cell death: Exposure to necrotic tumor cells, but not primary tissue cells or apoptotic cells, induces the maturation of immunostimulatory dendritic cells, *J. Exp. Med.*, **191,** 423-433.
38. Yonemura, Y., Takashima, T., Miwa, K., Miyazaki, I., Yamamoto, H. and Okamoto, H. (1984) Amelioration of *Diabetes mellitus* in partially depancreatized rats by poly(ADP-ribose) synthetase inhibitors: Evidence of islet B-cell regeneration, *Diabetes,* **33,** 401-404.
39. Terazono, K., Yamamoto, H., Takasawa, S., Shiga, K., Yonemura, Y., Tochino, Y. and Okamoto, H. (1988) A novel gene activated in regenerating islets, *J. Biol. Chem.,* **263,** 2111-2114.
40. Watanabe, T., Yonekura, H., Terazono, K., Yamamoto, H. and Okamoto, H. (1990) Complete nucleotide sequence of human *reg* gene and its expression in normal and tumoral tissues: The *reg* protein, pancreatic stone protein, and pancreatic thread protein are one and the same product of the gene, *J. Biol. Chem.,* **265,** 7432-7439.
41. Watanabe, T., Yonemura, Y., Yonekura, H., Suzuki, Y., Miyashita, H., Sugiyama, K., Moriizumi, S., Unno, M., Tanaka, O., Kondo, H., Bone, A.J., Takasawa, S. and Okamoto, H. (1994) Pancreatic beta-cell replication and amelioration of surgical diabetes by Reg protein, *Proc. Natl. Acad. Sci. USA,* **91,** 3589-3592.
42. Kobayashi , S., Akiyama, T., Nata, K., Abe, M., Tajima, M., Shervani, N. J., Unno, M., Matsuno, S., Sasaki, H., Takasawa, S. and Okamoto, H. (2000) Identification of a receptor for *Reg* (*Regenerating Gene*) protein, a pancreatic ß-cell regeneration factor, *J. Biol. Chem.,* **275,** 10723-10726.
43. Akiyama, T., Takasawa, S., Nata, N., Kobayashi, S., Abe, M., Shervani, N. J., Ikeda, T., Nakagawa, K., Unno, M., Matsuno, S. and Okamoto, H. (2001) Activation of *Reg* gene, a gene for insulin-producing β-cell regeneration: Poly (ADP-ribose) polymerase binds *Reg* promoter and regulates the transcription by autopoly(ADP-ribosyl)ation, Proc. *Natl. Acad. Sci. USA,* **98,** 48-53.
44. Moriizumi, S., Watanabe, T., Unno, M., Nakagawara, K., Suzuki, Y., Miyashita, H., Yonekura, H. and Okamoto, H. (1994) Isolation, structural determination and expression of a novel *reg* gene, human *reg Iß*, *Biochim. Biophys. Acta,* **1217,** 199-202.
45. Unno, M., Yonekura, H., Nakagawara, K., Watanabe, T., Miyashita, H., Moriizumi, S., Okamoto, H., Itoh, T. and Teraoka, H. (1993) Structure, chromosomal localization, and expression of mouse *reg* genes, *reg I* and *reg II*. A novel type of reg gene, reg II, exists in the mouse genome, *J. Biol. Chem.*, **268,** 15974-15982.
46. Bartoli, C., Gharib, B., Giorgi, D., Sansonetti, A., Dagorn, J. C. and Bergé-Lefranc, J. l. (1993) A gene homologous to the reg gene is expressed in the human pancreas, *FEBS Lett.*, **327,** 289-293.
47. Yonekura, H., Unno, M., Watanabe, T., Moriizumi, S., Suzuki, Y., Miyashita, H., Yonemura, Y., Sugiyama, K. and Okamoto, H. (1994) Structure and expression of *reg* gene family. In: Frontiers of Insulin Secretion and Pancreatic B-Cell Research, Edited by Flatt, P.R. and Lenzan S., pp. 581-588. London, Smith-Gorton.
48. Suzuki, Y., Watanabe, T., Unno, M., Moriizumi, S., Miyashita H., Yonekura, H. and Okamoto, H. (1994) Structure and expression of a novel rat *REG III* gene, *Gene,* **144,** 315-316.
49. Miyashita, H., Yonekura, H., Unno, M., Suzuki, Y., Watanabe, T., Moriizumi, S., Takasawa, S. and Okamoto, H. (1994) Characterization of the 5'-regulatory region of the rat *Reg I* gene, *Biochim. Biophys. Acta,* **1219,** 241-243.
50. Miyashita, H., Nakagawara, K., Mori, M., Narushima, Y., Noguchi, N., Moriizumi, S., Takasawa, S., Yonekura, H., Takeuchi, T. and Okamoto, H. (1995) Human *REG* family genes are tandemly ordered in a 95-kilobase region of chromosome 2p12, *FEBS Lett.,* **377,** 429-433.
51. Narushima, Y., Unno, M., Nakagawara, K., Mori, M., Miyashita, H., Suzuki, Y., Noguchi, N., Takasawa, S., Kumagai, T., Yonekura, H. and Okamoto, H. (1997) Structure, chromosomal localization and expression of mouse genes encoding type III Reg, *Reg IIIa, Reg IIIß, Reg IIIγ, Gene,* **185,** 159-168.
52. Okamoto, H. (1999) The *Reg* gene family and Reg proteins: With special attention to the regeneration of pancreatic ß-cells, *J. Hepatobiliary Pancreat. Surg.,* **6,** 254-262.

53. Abe, M., Nata, K., Akiyama, T., Shervani, N. J., Kobayashi, S., Tomioka-Kumagai, T., Ito, S., Takasawa, S. and Okamoto, H. (2000) Identification of a novel *Reg* family gene, *Reg IIIδ*, and mapping of all three types of *Reg* family genes in a 75-kilobase mouse genomic region, *Gene,* **246**, 111-122.

54. De La Monte, S. M., Ozturk, M. and Wands, J. R. (1990) Enhanced expression of an exocrine pancreatic protein in Alzheimer's disease and the developing human brain, *J. Clin. Invest.*, **86,** 1004-1013.

55. Iovanna, J., Orelle, B., Keim, V. and Dagorn, J. C. (1991) Messenger RNA sequence and expression of rat pancreatitis-associated protein, a lectin-related protein overexpressed during acute experimental pancreatitis, *J. Biol. Chem.*, **266,** 24664-24669.

56. Lasserre, C., Christa, L., Simon, M. T., Vernier, P. and Bréchot, C. (1992) A novel gene (HIP) activated in human primary liver cancer, *Cancer Res.*, **52,** 5089-5095.

57. Frigerio, J. M., Dusetti, N. J., Keim, V., Dagorn, J. C. and Iovanna, J. L. (1993) Identification of a second rat pancreatitis-associated protein: Messenger RNA cloning, gene structure, and expression during acute pancreatitis, *Biochemistry*, **32,** 9236-9241.

58. Frigerio, J. M., Dusetti, N. J., Garrido, P., Dagorn, J. C. and Iovanna, J. L. (1993) The pancreatitis associated protein III (PAP III), a new member of the PAP gene family, *Biochim. Biophys. Acta*, **1216**, 329-331.

59. Lasserre, C., Simon, M. T., Ishikawa, H., Diriong, S., Nguyen, V. C., Christa, L., Vernier, P. and Brechot, C. (1994) Structural organization and chromosomal localization of a human gene (HIP/PAP) encoding a C-type lectin overexpressed in primary liver cancer, *Eur. J. Biochem.*, **224,** 29-38.

60. Dusetti, N. J., Frigerio, J. M., Fox, M. F., Swallow, D. M., Dagorn, J. C. and Iovanna, J. L. (1994) Molecular cloning, genomic organization, and chromosomal localization of the human pancreatitis-associated protein (PAP) gene, *Genomics*, **19,** 108-114.

61. Katsumata, N., Chakraborty, C., Myal, Y., Schroedter, I. C., Murphy, L. J., Shiu, R. P. and Friesen, H. G. (1995) Molecular cloning and expression of peptide 23, a growth hormone-releasing hormone-inducible pituitary protein, *Endocrinology*, **136,** 1332-1339.

62. Rafaeloff, R., Pittenger, G. L., Barlow, S. W., Qin, X. F., Yan, B., Rosenberg, L., Duguid, W. P. and Vinik, A. I. (1997) Cloning and sequencing of the pancreatic islet neogenesis associated protein (INGAP) gene and its expression in islet neogenesis in hamsters, *J. Clin. Invest.*, **99,** 2100-2109.

63. Hartupee, J. C., Zhang, H., Bonaldo, M. F., Soares, M. B. and Dieckgraefe, B. K. (2001) Isolation and characterization of a cDNA encoding a novel member of the human regenerating protein family: Reg IV, *Biochim. Biophys. Acta*, **1518,** 287-293.

64. Livesey, J. F., O'Brien, A. J., Li, M., Smith, G. A., Murphy, J. L. and Hunt, P. S. (1997) A Schwann cell mitogen accompanying regeneration of motor neurons, *Nature*, **390,** 614-618.

65. Nishimune, H., Vasseur, S., Wiese, S., Birling, M. C., Holtmann, B., Sendtner, M., Iovanna, J. L. and Henderson, C. E. (2000) Reg-2 is a motoneuron neurotrophic factor and a signalling intermediate in the CNTF survival pathway, *Nature Cell Biol.*, **2,** 906-914.

66. Asahara, M., Mushiake, S., Shimada, S., Fukui, H., Kinoshita, Y., Kawakami, C., Watanabe, T., Tanaka, T., Ichikawa, A., Uchiyama, Y., Narushima, Y., Takasawa, S., Okamoto, H., Tohyama, M. and Chiba, T. (1996) *Reg* gene expression is increased in rat gastric enterochromaffin-like cells following water immersion stress, *Gastroenterology,* **111,** 45-55.

67. Fukui, H., Kinoshita, Y., Maekawa, T., Okada, A., Waki, S., Hassan, S., Okamoto, H. and Chiba, T. (1998) Regenerating gene protein may mediate gastric mucosal proliferation induced by hypergastrinemia in rats, *Gastroenterology,* **115,** 1483-1493.

68. Kazumori, H., Ishirara, S., Hoshino, E., Kawashima, K., Moriyama, N., Suetsugu, H., Sato, H., Adachi, K., Fukuda, R., Watanabe, M., Takasawa, S., Okamoto, H., Fukui, H., Chiba, T. and Kinoshita, Y. (2000) Neutrophil chemoattractant-2ß regulates the expression of the Reg gene in injured gastric mucosa in rats, *Gastroenterology,* **119,** 1610-1622.

69. Ashcroft, F. M., Harrison, D. E. and Ashcroft, S. J. H. (1984) Glucose induced closure of single potassium channels in isolated rat pancreatic ß-cells, *Nature*, **312,** 446-448.

70. Takasawa, S., Nata, K., Yonekura, H. and Okamoto, H. (1993) Cyclic ADP-ribose in insulin secretion from pancreatic ß cells, *Science*, **259,** 370-373.

71. Takasawa, S., Akiyama, T., Nata, K., Kuroki, M., Tohgo, A., Noguchi, N., Kobayashi, K., Kato, I., Katada, T. and Okamoto, H. (1998) Cyclic ADP-ribose and inositol 1,4,5-trisphosphate as alternate second messengers for intracellular $Ca^{2+}$ mobilization in normal and diabetic ß-cells, *J. Biol. Chem.,* **273,** 2497-2500.

72. Takasawa, S., Tohgo, A., Noguchi, N., Koguma, T., Nata, K., Sugimoto, T., Yonekura, H. and Okamoto, H. (1993) Synthesis and hydrolysis of cyclic ADP-ribose by human leukocyte antigen CD38 and inhibition of the hydrolysis by ATP, *J. Biol. Chem.,* **268,** 26052-26054.

73. Koguma, T., Takasawa, S., Tohgo, A., Karasawa, T., Furuya, Y., Yonekura, H. and Okamoto, H. (1994) Cloning and characterization of cDNA encoding rat ADP-ribosyl cyclase/cyclic ADP-ribose hydrolase (homologue to human CD38) from islets of Langerhans, *Biochim. Biophys. Acta,* **1223,** 160-162.

74. Howard, M., Grimaldi, J. C., Bazan, J. F., Bazan, J. F., Lund, F. E., Santos-Argumedo, L., Parkhouse, R. M., Walseth, T. F. and Lee, H. C. (1993) Formation and hydrolysis of cyclic ADP-ribose catalyzed by lymphocyte antigen CD38, *Science*, **262,** 1056-1059.

75. Zocchi, E., Franco, L., Guida, L., Benatti, U., Bargellesi, A., Malavasi, F., Lee, H. C. and De Flora, A. (1993) A single protein immunologically identified as CD38 displays $NAD^+$ glycohydrolase, ADP-ribosyl cyclase and cyclic ADP-ribose hydrolase activities at the outer surface of human erythrocytes, *Biochem. Biophys. Res. Commun.*,

**196,** 1459-1465.

76. Tohgo, A., Munakata, H., Takasawa, S., Nata, K., Akiyama, T., Hayashi, N. and Okamoto, H. (1997) Lysine 129 of CD38 (ADP-ribosyl cyclase/cyclic ADP-ribose hydrolase) participates in the binding of ATP to inhibit the cyclic ADP-ribose hydrolase, *J. Biol. Chem.,* **272,** 3879-3882.

77. Tohgo, A., Takasawa, S., Noguchi, N., Koguma, T., Nata, K., Sugimoto, T., Furuya, Y., Yonekura, H. and Okamoto, H. (1994) Essential Cysteine Residues for Cyclic ADP-ribose Synthesis and Hydrolysis by CD38, *J. Biol. Chem.,* **269,** 28555-28557.

78. Okamoto, H. and Takasawa, S. (2001) CD38. In: Encyclopedia of Molecular Medicine, Edited by Creighton, T. E., in press, New York, John Wiley & Sons, Inc.

79. Mészáros, L. G., Bak, J. and Chu, A. (1993) Cyclic ADP-ribose as an endogenous regulator of the non-skeletal type ryanodine receptor $Ca^{2+}$ channel, *Nature*, **364,** 76-79.

80. Mitchell, K. J., Pinton, P., Varadi, A., Tacchetti, C., Ainscow, E. K., Pozzan, T., Rizzuto, R. and Rutter, G. A. (2001) Dense core secretory vesicles revealed as a dynamic $Ca^{2+}$ store in neuroendocrine cells with a vesicle-associated membrane protein aequorin chimaera, *J. Cell Biol.*, **155,** 41-51.

81. Noguchi, N., Takasawa, S., Nata, K., Tohgo, A., Kato, I., Ikehata, F., Yonekura, H. and Okamoto, H. (1997) Cyclic ADP-ribose binds to FK506-binding protein 12.6 to release $Ca^{2+}$ from islet microsomes, *J. Biol. Chem.,* **272,** 3133-3136.

82. Fukao, K., Ochiai, T., Takahashi, K., Endo, T., Yokoyama, I., Uchida, K., Oshima, S., Ishibashi, M., Takahara, S., Iwasaki, Y., Ota, K., Takagi, H. and Sonoda, T. (1994) A late phase II study of FK506 (Tacrolimus) on kidney transplantation, *Jps. J. Transplantation*, **29,** 614-631.

83. Pirsch, J. D., Miller, J., Deierhoi, M. H., Vincenti, F. and Filo, R. S. (1997) A comparison of tacrolimus (FK506) and cyclosporine for immunosuppression after cadaveric renal transplantation: FK506 Kidney Transplant Study Group, *Transplantation*, **63,** 977-983.

84. Takasawa, S., Ishida, A., Nata, K., Nakagawa, K., Noguchi, N., Tohgo, A., Kato, I., Yonekura, H., Fujisawa, H. and Okamoto, H. (1995) Requirement of calmodulin-dependent protein kinase II in cyclic ADP-ribose-mediated intracellular $Ca^{2+}$ mobilization, *J. Biol. Chem.,* **270,** 30257-30259.

85. Kato, I., Suzuki, Y., Akabane, A., Yonekura, H., Tanaka, O., Kondo, H., Takasawa, S., Yoshimoto, T. and Okamoto, H. (1996) Enhancement of glucose-induced insulin secretion in transgenic mice overexpressing human VIP gene in pancreatic ß cells, *Ann. NY Acad. Sci.,* **805,** 232-243.

86. Kato, I., Yamamoto, Y., Fujimura, M., Noguchi, N., Takasawa, S. and Okamoto, H. (1999) CD38 disruption impairs glucose-induced increases in cyclic ADP-ribose, $[Ca^{2+}]i$ and insulin secretion, *J. Biol. Chem.,* **274,** 1869-1872.

87. Kato, I., Takasawa, S., Akabane, A., Tanaka, O., Abe, H., Takamura, T., Suzuki, Y., Nata, K., Yonekura, H., Yoshimoto, T. and Okamoto, H. (1995) Regulatory role of CD38 (ADP-ribosyl cyclase/cyclic ADP-ribose hydrolase) in insulin secretion by glucose in pancreatic ß cells: Enhanced insulin secretion in CD38 transgenic mice, *J. Biol. Chem.,* **270,** 30045-30050.

88. Nakagawara, K., Mori, M., Takasawa, S., Nata, K., Takamura, T., Berlova, A., Tohgo, A., Karasawa, T., Yonekura, H., Takeuchi, T. and Okamoto, H. (1995) Assignment of the CD38 gene encoding human leukocyte antigen CD38 (ADP-ribosyl cyclase/cyclic ADP-ribose hydrolase) to chromosome 4p15, *Cytogenet. Cell Genet.,* **69,** 38-39.

89. Nata, K., Takamura, T., Karasawa, T., Kumagai, T., Hashioka, W., Tohgo, A., Yonekura, H., Takasawa, S., Nakamura, S. and Okamoto, H. (1997) Human gene encoding CD38 (ADP-ribosyl cyclase/cyclic ADP-ribose hydrolase): organization, nucleotide sequence and alternative splicing, *Gene,* **186,** 285-292.

90. Yagui, K., Shimada, F., Miura, M., Hashimoto, N., Suzuki, Y., Tokuyama, Y., Nata, K., Tohgo, A., Ikehata, F., Takasawa, S., Okamoto, H., Makino, H., Saito, Y. and Kanatsuka, A. (1998) A missense mutation in the CD38 gene, a novel factor for insulin secretion: Association with Type II diabetes mellitus in Japanese subjects and evidence of abnormal function when expressed in vitro, *Diabetologia,* **41,** 1024-1028.

91. Ikehata, F., Satoh, J., Nata, K., Tohgo, A., Nakazawa, T., Kato, I., Kobayashi, S., Akiyama, T., Takasawa, S., Toyota, T. and Okamoto, H. (1998) Autoantibodies against CD38 (ADP-ribosyl cyclase/cyclic ADP-ribose hydrolase) which impair glucose-induced insulin secretion in non-insulin dependent diabetes patients, *J. Clin. Invest.,* **102,** 395-401.

92. Pupilli, C., Giannini, S., Marchetti, P., Lupi, R., Antonelli, A., Malavasi, F., Takasawa, S., Okamoto, H. and Ferrannini, E. (1999) Autoanitibodies to CD38 (ADP-ribosyl cyclase/cyclic ADP-ribose hydrolase) in Caucasian patients with diabetes: Effects on insulin release from human islets, *Diabetes,* **48,** 2309-2315.

93. Mallone, R., Ortolan, E., Baj, G., Funaro, A., Giunti, S., Lillaz, E., Saccucci, F., Cassader, M., Cavallo-Perin, P. and Malavasi, F. (2001) Autoantibody response to CD38 in Caucasian patients with type 1 and type 2 diabetes: immunological and genetic characterization, *Diabetes*, **50,** 752-762.

94. Antonelli, A., Baj, G., Marchetti, P., Fallahi, P., Surico, N., Pupilli, C., Malavasi, F. and Ferrannini, E. (2001) Human anti-CD38 autoantibodies raise intracellular calcium and stimulate insulin release in human pancreatic islets, *Diabetes*, **50,** 985-991.

95. Berridge, M. J. and Irvine, R. F. (1984) Inositol trisphosphate, a novel second messenger in cellular signal transduction, *Nature*, **312,** 315-321.

96. Islam, M. S., Larsson, O., Berggren, P.-O., Takasawa, S., Nata, K., Yonekura, H., Okamoto, H. and Galione, A. (1993) Cyclic ADP-ribose in ß cells, *Science*, **262,** 584-586.

97. Rutter, G. A., Theler, J.-M. and Wollheim, C. B. (1994) $Ca^{2+}$ stores in insulin-secreting cells: lack of effect of cADP

ribose, *Cell Calcium*, **16**, 71-80.

98. Webb, D.-L., Islam, M. S., Efanov, A. M., Brown, G., Kohler, M., Larsson, O., Berggren, P.-O. (1996) Insulin exocytosis and glucose-mediated increase in cytoplasmic free $Ca^{2+}$ concentration in the pancreatic β-cell are independent of cyclic ADP-ribose, *J. Biol. Chem.*, **271**, 19074-19079.

99. Islam, M. S. and Berggren, P. O. (1997) Cyclic ADP-ribose and pancreatic beta cell: where do we stand?, *Diabetologia*, **40**, 1480-1484.

100. Okamoto, H., Takasawa, S. and Nata, K. (1997) The CD38—cyclic ADP-ribose signaling system in insulin secretion: molecular basis and clinical implications, *Diabetologia,* **40**, 1485-1491.

101. Walseth, T. F. and Lee, H. C. (1993) Synthesis and characterization of antagonists of cyclic-ADP-ribose-induced $Ca^{2+}$ release, *Biochim. Biophys. Acta*, **1178,** 235-242.

102. An, N. H., Han, M. K., Um, C., Park, B. H., Park, B. J., Kim, H. K. and Kim, U. H. (2001) Significance of ectocyclase activity of CD38 in insulin secretion of mouse pancreatic islet cells, *Biochem. Biophys. Res. Commun.*, **282,** 781-786.

103. Galione, A. (1993) Cyclic ADP-ribose: A new way to control calcium, *Science*, **259**, 325-326.

104. Sasaki, T., Shimura, S., Takasawa, S., Nagaki, M., Satoh, M., Okamoto, H. and Shirato, K. (1993) Cyclic ADP-ribose, a candidate for a novel $Ca^{2+}$-mobilizing second messenger, induced $Ca^{2+}$-dependent current responses in airway submucosal gland cells, *Am. Rev. Resp. Dis.*, **147,** A936.

105. Hua, S.-Y., Tokimasa, T., Takasawa, S., Furuya, Y., Nohmi, M., Okamoto, H. and Kuba, K. (1994) Cyclic ADP-ribose modulates $Ca^{2+}$ release channels for activation by physiological $Ca^{2+}$ entry in bullfrog sympathetic neurons, *Neuron*, **12**, 1073-1079.

106. Thorn, P., Gerashimenko, O. and Petersen, O. H. (1994) Cyclic ADP-ribose regulation of ryanodine receptors involved in agonist evoked cytosolic $Ca^{2+}$ oscillations in pancreatic acinar cells, *EMBO J.*, **13**, 2038-2043.

107. Lee, H. C., Aarhus, R., Graeff, R., Gurnack, M. E. and Walseth, T. F. (1994) Cyclic ADP ribose activation of the ryanodine receptor is mediated by calmodulin, *Nature*, **370**, 307-309.

108. Tanaka, Y. and Tashjian, A. H. Jr. (1995) Calmodulin is a selective mediator of $Ca^{2+}$-induced $Ca^{2+}$ release via the ryanodine receptor-like $Ca^{2+}$ channel triggered by cyclic ADP-ribose, *Proc. Natl. Acad. Sci. USA*, **92,** 3244-3248.

109. Higashida, H., Robbins, J., Egorova, A., Noda, M., Taketo, M., Ishizaka, N., Takasawa, S., Okamoto, H. and Brown, D. A. (1995) Nicotinamide-adenine dinucleotide regulates muscarinic receptor-coupled K+(M) channels in rodent NG108-15 cells, *J. Physiol.*, **482**, 317-323.

110. Allen, G. J., Muir, S. R. and Sanders, D. (1995) Release of $Ca^{2+}$ from individual plant vacuoles by both InsP3 and cyclic ADP-ribose, *Science*, **268**, 735-737.

111. Kuemmerle, J. F. and Makhlouf, G. M. (1995) Agonist-stimulated cyclic ADP ribose: Endogenous modulator of $Ca^{2+}$-induced $Ca^{2+}$ release in intestinal longitudinal muscle, *J. Biol. Chem.*, **270,** 25488-25494.

112. Gromada, J., Jørgensen, T. D. and Dissing, S. (1995) Cyclic ADP-ribose and inositol 1,4,5-trisphosphate mobilizes $Ca^{2+}$ from distinct intracellular pools in permeabilized lacrimal acinar cells, *FEBS Lett.*, **360,** 303-306.

113. Rakovic, S., Galione, A., Ashamu, G. A., Potter, B. V. L. and Terrar, D. A. (1996) A specific cyclic ADP-ribose antagonist inhibits cardiac excitation-contraction coupling, *Curr. Biol.*, **6,** 989-996.

114. Ebihara, S., Sasaki, T., Hida, W., Kikuchi, Y., Oshiro, T., Shimura, S., Takasawa, S., Okamoto, H., Nishiyama, A., Akaike, N. and Shirato, K. (1997) Role of cyclic ADP-ribose in ATP-activated potassium currents in alveolar macrophages, *J. Biol. Chem.*, **272,** 16023-16029.

115. Yamaki, H., Morita, K., Kitayama, S., Imai, Y., Itadani, K., Akagawa, Y. and Doi, T. (1998) Cyclic ADP-ribose induces $Ca^{2+}$ release from caffeine-insensitive $Ca^{2+}$ pools in canine salivary gland cells, *J. Dental Res.*, **77,** 1807-1816.

116. Prakash, Y. S., Kannan, M. S., Walseth, T. F. and Sieck, G. C. (1998) Role of cyclic ADP-ribose in the regulation of $[Ca^{2+}](i)$ in porcine tracheal smooth muscle, *Am. J. Physiol.*, **43,** C1653-C1660.

117. Mothet, J. P., Fossier, P., Meunier, F. M., Stinnakre, J., Tauc, L., Baux, G. (1998) Cyclic ADP-ribose and calcium-induced calcium release regulate neurotransmitter release at a cholinergic synapse of *Aplysia*, *J. Physiol.*, **507,** 405-414.

118. Li, P. L., Zou, A. P. and Campbell, W. B. (1998) Regulation of K-Ca-channel activity by cyclic ADP-ribose and ADP-ribose in coronary arterial smooth muscle, *Am. J. Physiol.*, **44,** H1002-H1010.

119. Sun, L., Adebanjo, O. A., Moonga, B.S., Corisdeo, S., Anandatheerthavarada, H. K., Biswas, G., Arakawa, T., Hakeda, Y., Koval, A., Sodam, B., Bevis, P. J., Moser, A. J., Lai, F. A., Epstein, S., Troen, B. R., Kumegawa, M. and Zaidi, M. (1999) CD38/ADP-ribosyl cyclase: A new role in the regulation of osteoclastic bone resorption, *J. Cell Biol.*, **146,** 1161-1172.

120. Reyes-Harde, M., Potter, B. V., Galione, A. and Stanton, P. K. (1999) Induction of hippocampal LTD requires nitric-oxide-stimulated PKG activity and Ca(2+) release from cyclic ADP-ribose-sensitive stores, *J. Neurophysiol.*, **82,** 1569-1576.

121. Rakovic, S., Cui, Y., Iino, S., Galione, A., Ashamu, G. A., Potter, B. V. and Terrar, D. A. (1999) An antagonist of cADP-ribose inhibits arrhythmogenic oscillations of intracellular $Ca^{2+}$ in heart cells, *J. Biol. Chem.*, **274,** 17820-17827.

122. Inngjerdingen, M., Al-Aoukaty, A., Damaj, B. and Maghazachi, A. A. (1999) Differential utilization of cyclic ADP-ribose pathway by chemokines to induce the mobilization of intracellular calcium in NK cells, *Biochem. Biophys. Res. Commun.*, **262,** 467-472.

123. Guse, A. H., da Silva, C. P., Berg, I., Skapenko, A. L., Weber, K., Heyer, P., Hohenegger, M., Ashamu, G. A., Schulze-Koops, H., Potter, B. V. and Mayr, G. W. (1999) Regulation of calcium signalling in T lymphocytes by the second messenger cyclic ADP-ribose, *Nature*, **398,** 70-73.

124. Khoo, K. M., Han, M. K., Park, J. B., Chae, S. W., Kim, U. H., Lee, H. C., Bay, B. H. and Chang, C. F. (2000) Localization of the cyclic ADP-ribose dependent calcium signaling pathway in hepatocyte nucleus, *J. Biol. Chem.* **275,** 24807-24817.

125. Han, M. K., Cho, Y. S., Kim, Y. S., Yim, C. Y. and Kim, U. H. (2000) Interaction of two classes of ADP-ribose transfer reactions in immune signaling, *J. Biol. Chem.*, **275,** 20799-20805.

126. Fukushi, Y., Kato, I., Takasawa, S., Sasaki, S., Ong, B. H., Ohsaga, A., Sato, K., Shirato, K., Okamoto, H. and Maruyama, Y. (2001) Identification of cyclic ADP-ribose-dependent mechanisms in pancreatic muscarinic $Ca^{2+}$ signaling using CD38 knockout mice, *J. Biol. Chem.,* **276,** 649-655.

127. Partida-Sánchez, S., Coockayne, D. A., Monard, S., Jacobson, E. L., Oppenheimer, N., Garvy, B., Kusser, K., Goodrich, S., Howard, M., Harmsen, A., Randall, T. D. and Lund, F. E. (2001) Cyclic ADP-ribose production by CD38 regulates intracellular calcium release, extracellular calcium influx and chemotaxis in neutrophils and is required for bacterial clearance *in vivo*, *Nature Med.*, **7,** 1209-1216.

128. Higashida, H., Yokoyama, S., Hashii, M., Taketo, M., Higashida, M., Takayasu, T., Ohshima, T., Takasawa, S., Okamoto, H. and Noda, M. (1997) Muscarinic receptor-mediated dual regulation of ADP-ribosyl cyclase in NG108-15 neuronal cell membranes analyzed by thin layer chromatography, *J. Biol. Chem.,* **272,** 31272-31277.

